# Cytoskeletal protein flightless I inhibits apoptosis, enhances tumor cell invasion and promotes cutaneous squamous cell carcinoma progression

**DOI:** 10.18632/oncotarget.5536

**Published:** 2015-10-19

**Authors:** Zlatko Kopecki, Gink N. Yang, Jessica E. Jackson, Elizabeth L. Melville, Matthew P. Cal1ey, Dedee F. Murrell, Ian A. Darby, Edel A. O'Toole, Michael S. Samuel, Allison J. Cowin

**Affiliations:** ^1^ Regenerative Medicine, Future Industries Institute, University of South Australia, Adelaide, South Australia, Australia; ^2^ Centre for Cutaneous Research, Blizard Institute, Barts and the London School of Medicine and Dentistry, Queen Mary University of London, London, United Kingdom; ^3^ Department of Dermatology, St. George Hospital and University of New South Wales, Sydney, New South Wales, Australia; ^4^ School of Medical Sciences, RMIT University, Melbourne, Victoria, Australia; ^5^ Centre for Cancer Biology, an alliance between SA Pathology and the University of South Australia, Australia

**Keywords:** flightless, squamous cell carcinoma, cell invasion, skin cancer

## Abstract

Flightless I (Flii) is an actin remodeling protein that affects cellular processes including adhesion, proliferation and migration. In order to determine the role of Flii during carcinogenesis, squamous cell carcinomas (SCCs) were induced in *Flii* heterozygous (*Flii^+/−^*), wild-type and *Flii* overexpressing (*Flii^Tg/Tg^*) mice by intradermal injection of 3-methylcholanthrene (MCA). Flii levels were further assessed in biopsies from human SCCs and the human SCC cell line (MET-1) was used to determine the effect of Flii on cellular invasion. Flii was highly expressed in human SCC biopsies particularly by the invading cells at the tumor edge. *Flii^Tg/Tg^* mice developed large, aggressive SCCs in response to MCA. In contrast *Flii^+/−^* mice had significantly smaller tumors that were less invasive. Intradermal injection of Flii neutralizing antibodies during SCC initiation and progression significantly reduced the size of the tumors and, *in vitro*, decreased cellular sphere formation and invasion. Analysis of the tumors from the *Flii* overexpressing mice showed reduced caspase I and annexin V expression suggesting Flii may negatively regulate apoptosis within these tumors. These studies therefore suggest that Flii enhances SCC tumor progression by decreasing apoptosis and enhancing tumor cell invasion. Targeting Flii may be a potential strategy for reducing the severity of SCCs.

## INTRODUCTION

Cutaneous squamous cell carcinomas (SCCs) are invasive, recurrent and in some cases potentially metastatic [1, 2]. Identifying new target proteins that inhibit cancer cell invasion and stimulate tumor apoptosis could lead to new treatments to reduce the severity of SCCs. Dynamic actin remodeling, alterations in cell morphology, apoptosis, adhesion and motility are all crucial aspects of tumor cell invasion and metastasis [3–6]. It has long been known that the actin cytoskeleton is deregulated in tumor cells and the cytoskeleton has been linked to aberrant tumor-microenvironment interactions in cutaneous SCC [4–8]. Flightless I (Flii), a multifunctional actin remodeling protein, has been identified as a tumor promoter with transcriptional activity in colorectal, breast and hepatocellular carcinoma cell lines [9, 10]. Flii is a member of the gelsolin family of cytoskeletal proteins that controls actin by severing pre-existing filaments and/or capping filament ends to enable filament reassembly into new cytoskeletal structures [11, 12]. Numerous cellular activities have been shown to involve Flii including the regulation of transcription via co-activation of nuclear hormone receptors [13–15], type I collagen gene [15], PPARy [16], and the regulation of β-catenin-dependent transcription [12]. Flii suppresses proliferation via interactions with calmodulin-dependent protein kinase type II [17], inflammation, and cytokine production via effects on Toll-like receptor signaling [18–20]. In addition, Flii effects on inflammation are facilitated by inhibition of caspase activation and suppression of IL-1beta maturation and caspase-1 mediated apoptosis [21, 22]. Actin polymerization can be inhibited by Flii as it forms a “cap” to prevent further elongation of the actin polymers which are necessary for cellular locomotion [23]. Flii stabilizes focal adhesions and over-expression leads to a decrease in the hemi-desmosome component CD151, laminin-332 and altered expression of laminin integrin receptors α3β1 and α6β4 [24]. Increased expression of Flii also impairs focal adhesion turnover in a Rac1 reliant manner and enhances formation of fibrillar adhesions [25] while promoting reduced cell spreading and elongated protrusion formation enabling collagen remodeling [26]. Through its bipartite domain structure, Flii is able to bind with numerous structural and signaling proteins at the basement membrane and transduce cell signaling events into cytoskeletal remodeling, inhibiting integrin facilitated stable anchoring contacts and active turnover of focal adhesions [25]. Interestingly Flii is also secreted in response to tissue injury [27]. Its extracellular function has been linked to regulation of tissue inflammation and reducing its expression using the application of neutralizing antibodies results in improved healing of wounds [28–31]. Indeed, the association between regulators of wound healing, inflammation and cancer have been well established [32, 33]. In addition, the function of Flii as a nuclear receptor co-activator and its involvement in different cell signaling pathways whereby it alters extracellular matrix composition and promotes tissue scarring suggests a potential promoting activity for Flii in tumor growth and spread [34–36]. Furthermore, a number of studies have shown that the expression levels of actin remodeling proteins oscillate during tumor progression and that therapies targeting those proteins may inhibit cell motility and invasion [3, 9, 37, 38].

In this study, we have investigated the effect of Flii on SCC development and determined the effect of reducing Flii both genetically and using Flii-neutralizing antibodies (FnAb) on SCC development and tumor progression.

## RESULTS

### Human and murine cutaneous SCCs have elevated levels of Flii

**Figure 1:**
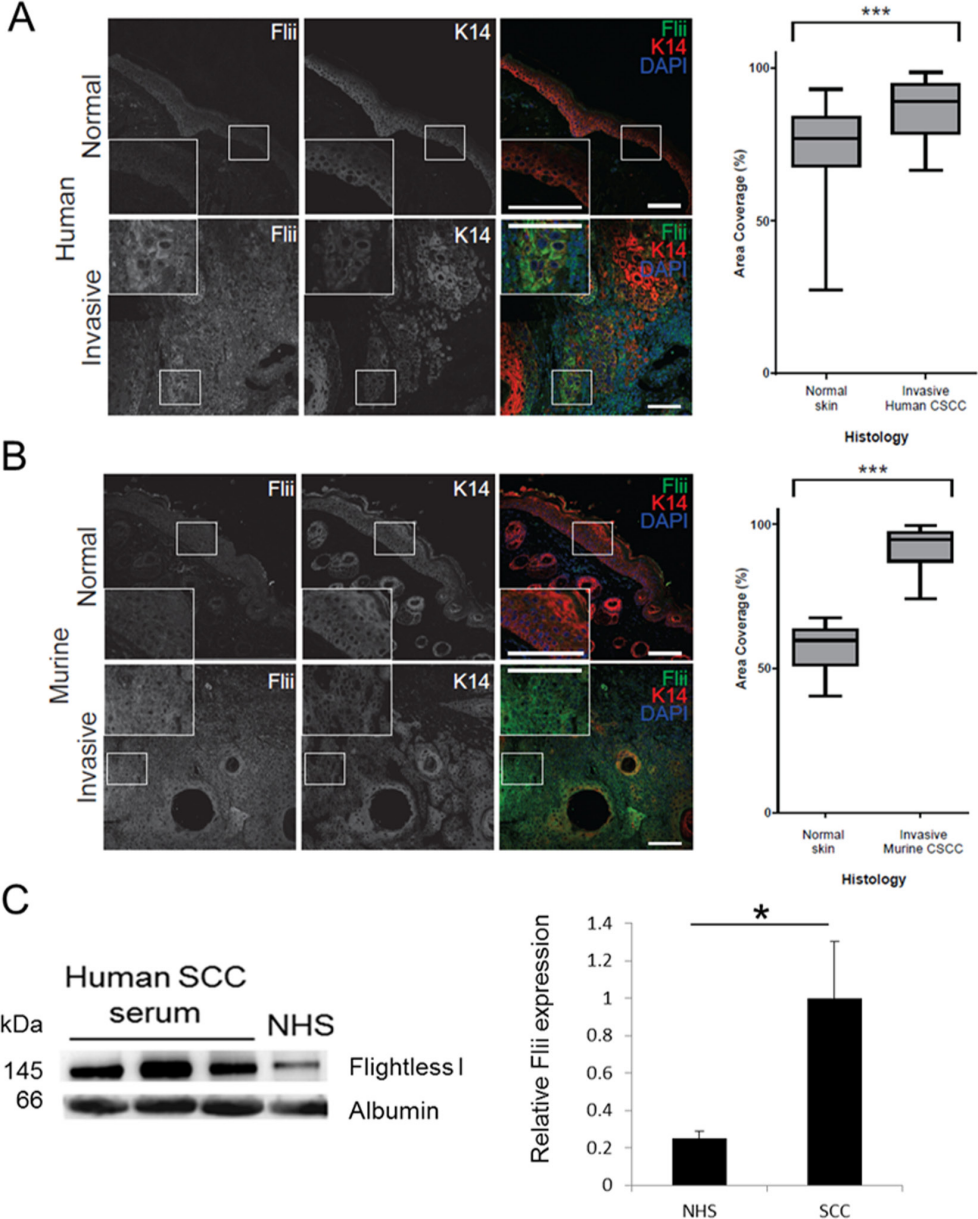
Flightless protein levels are increased in invasive cutaneous SCC **A–B.** Immunofluorescence analysis of Flii (greyscale in signal channel images and green in Merge) expression in normal margin skin and invasive cutaneous SCC of human (A, *N* = 21) or murine (B, *N* = 5) origin. Cytokeratin 14 (K14) immunofluorescence (greyscale in signal channel images and red in Merge) marks keratinocytes. Box and whisker plots show the median, range and quartiles of area coverage analysis carried out on the green channel to quantify Flii expression. Statistical significance was calculated using the Mann-Whitney test with Dunnet's post hoc test. *** *P* < 0.001. Magnification ×10 Insert ×20. Scale Bar = 100 μm **C.** Levels of secreted Flii were also examined in serum of three SCC patients. Flii is increased in serum of SCC patients (*n* = 3) compared to normal human serum (NHS) control. Albumin was used as a loading control. Graphical representation of Flii protein expression determined by densitometry. Figure is representative of three independent experiments. Mean +/− SD *p < 0.05.

Human SCC biopsies were collected from patients with invasive cutaneous SCC following pathologist diagnosis. They showed pathological features of invasive cutaneous SCC including anastomosing nests and strands of atypical keratinocytes originating in the epidermis and infiltrating deep into the dermis with evidence of intracellular bridges, parakeratosis and horn pearl formation (Supplementary Figure S1). Flii expression was significantly elevated in the human invasive cutaneous SCC compared to normal human skin with positive staining observed in the invading keratinocytes, surrounding tumor stroma and outer hyperkeratotic layer of the SCC nodules present in the deep dermis (Figure 1A). Flii expression was also observed in murine invasive cutaneous SCC samples with elevated Flii staining observed in both invading keratinocytes and dermal fibroblasts surrounding the tumor when compared to normal control skin (Figure 1B). Serum samples were collected from three patients with SCC and the level of secreted Flii determined. Compared to normal, commercially available, human serum, Flii levels were significantly elevated with an increase of 74.87% +/− 30.19% observed (Figure 1C).

### Severe SCCs develop when flii levels are elevated

**Figure 2:**
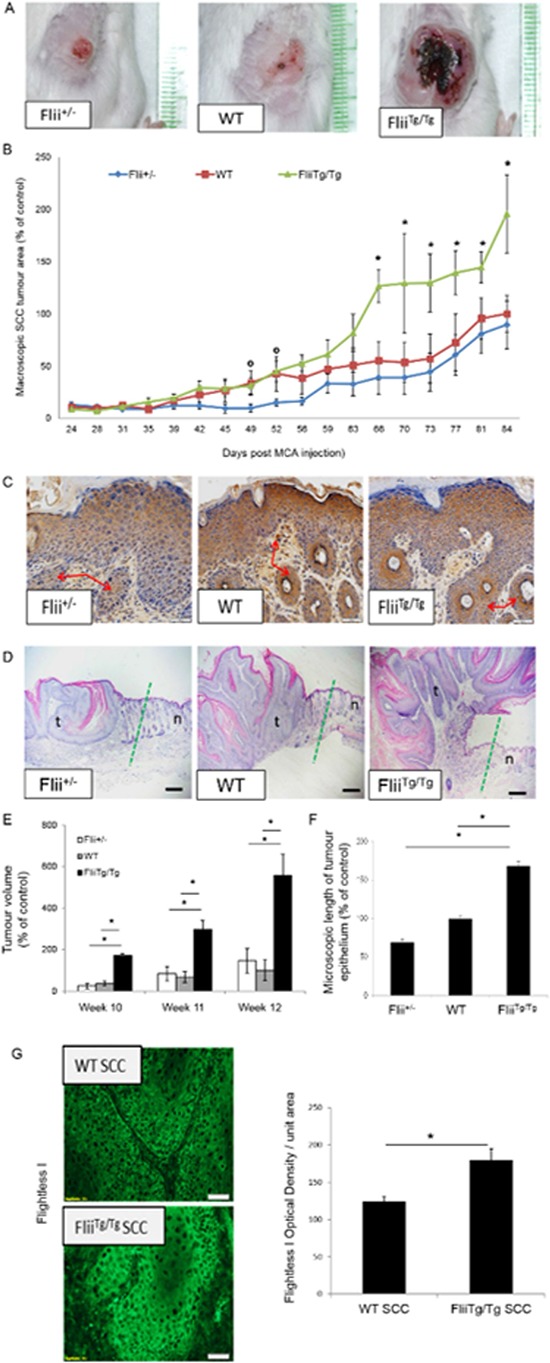
Overexpression of Flii results in severe SCC development **A–B.** Using the MCA induced model of SCC in *Flii^+/−^*, WT and *Flii^Tg/Tg^* mice we demonstrate that over-expression of *Flii* leads to quicker onset and more severe progression of necrotic and ulcerated SCC tumor lesions with significantly larger tumor area (*n* = 12/genotype) Mean +/− SD * and ° = *P* < 0.05. * denotes significance vs *Flii^Tg/Tg^* and ° denotes significance vs *Flii^+/−^*. **C.** Epithelial origin of invading cells and hyperkeratotic nodules (red arrows) present in the dermis of *Flii^+/−^*, WT and *Flii^Tg/Tg^* SCC induced mice was confirmed with positive cytokeratin staining in all samples. **D–F.** Representative images of tumor margins (dotted green line) in *Flii^+/−^*, WT and *Flii^Tg/Tg^* mice and macroscopic analysis of tumor volume and microscopic analysis of length of tumor epithelium revealing significantly larger tumors in *Flii* over-expressing mice. (*n* = 12/genotype) Mean +/− SD *P < 0.05. t = tumor n = normal skin **G.** Representative images of increased Flii expression in tumors of *Flii^Tg/Tg^* mice compared to wild-type controls. (*n* = 12/genotype) Mean +/− SD *P < 0.05. Magnification ×20 and ×10. Scale Bar = 100 μm and 500 μm.

Cutaneous SCCs were induced in *Flii^+/−^*, wild-type and *Flii^Tg/Tg^* mice by a single intradermal injection of MCA. *Flii^+/−^* mice with only one copy of the gene have half the amount of Flii protein compared to WT while *Flii^Tg/Tg^* with two extra copies of the gene have 1.52 fold increased levels of Flii protein in the skin [39]. *Flii* heterozygous mice had significantly smaller tumor areas compared to wild-type counterparts at day 49 and 52 of the experiment, with both groups of mice showing superficial moderate to well differentiated tumor papules, hyperchromatic nuclei and epidermal atypia (Figure 2A–2D). In contrast, from day 66 onwards, tumors on the *Flii^Tg/Tg^* mice greatly increased in both area (≤ 2 fold increase) (Figure 2A–2B) and volume (≤ 3 fold increase) (Figure 2E) compared to WT and *Flii^+/−^*mice. At day 84 when the trial was completed, the tumors were assessed histologically with larger, well differentiated tumors with extracellular keratin pearls and intercellular bridges observed in the *Flii^Tg/Tg^* mice compared to both wild-type and *Flii^+/−^* mice (Figure 2C–2D). Flii expression was increased by 31.03% +/− 8.25% in SCCs of *Flii^Tg/Tg^* mice compared to SCC in wild-type mice (Figure 2G). The severe development of SCC in *Flii^Tg/Tg^* mice was confirmed using cytokeratin staining with cytokeratin positive hyperkeratotic nodules and significantly longer tumor epithelium observed in *Flii^Tg/Tg^*compared to wild-type and *Flii^+/−^* mice (Figure 2C, 2D and 2F).

### Tumors in *Flii^Tg/Tg^* mice have decreased markers of apoptosis

**Figure 3:**
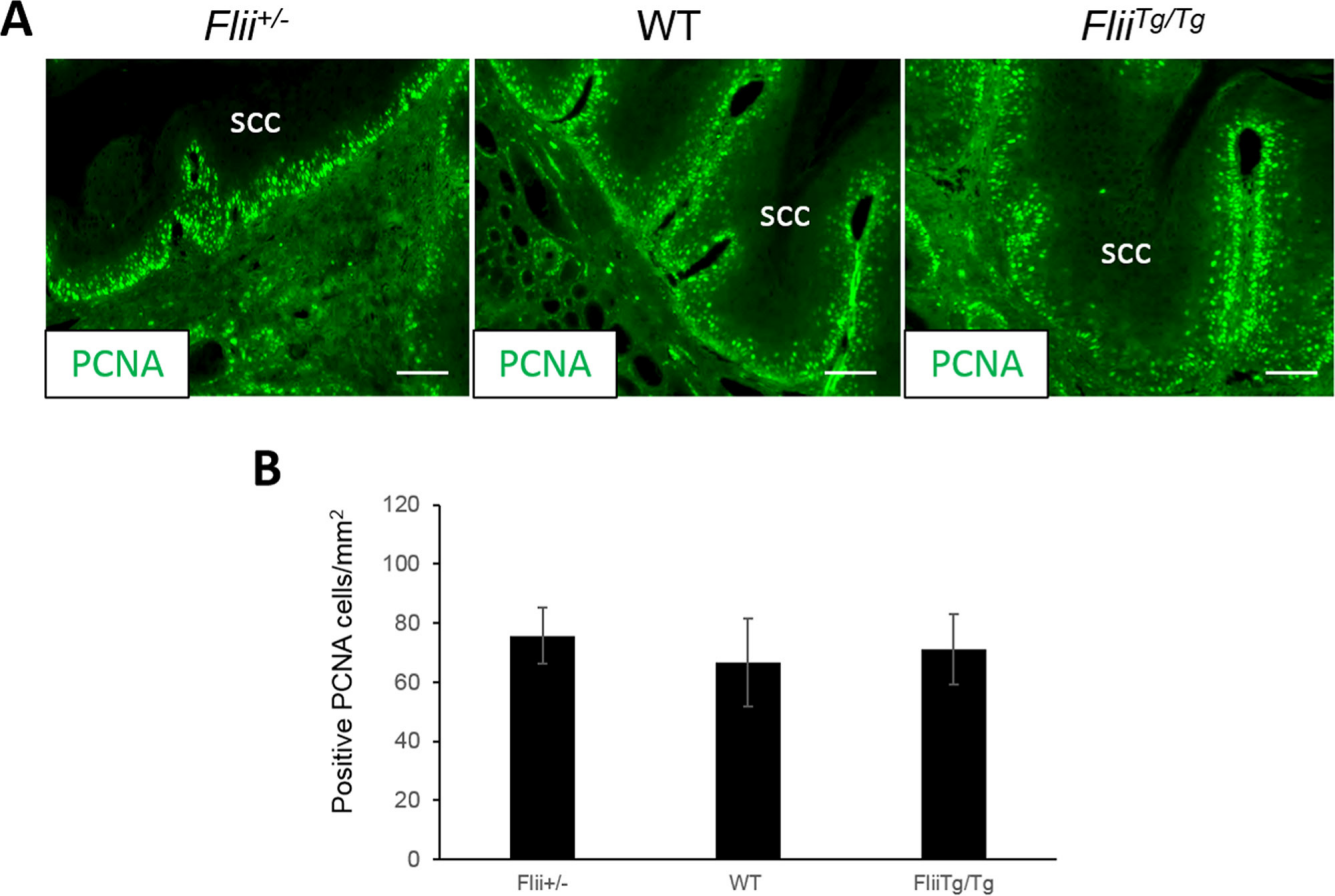
Flii does not influence the proliferation of cutaneous SCC tumors **A–B.** Cutaneous SCC tumors in *Flii^+/−^*, WT and *Flii^Tg/Tg^* mice were stained for PCNA and number of positive proliferating cells analysed showing no effect of Flii on tumor proliferation. (*n* = 12/genotype). Magnification × 20. Scale Bar = 100 μm. Mean +/− SD *P < 0.05.

**Figure 4:**
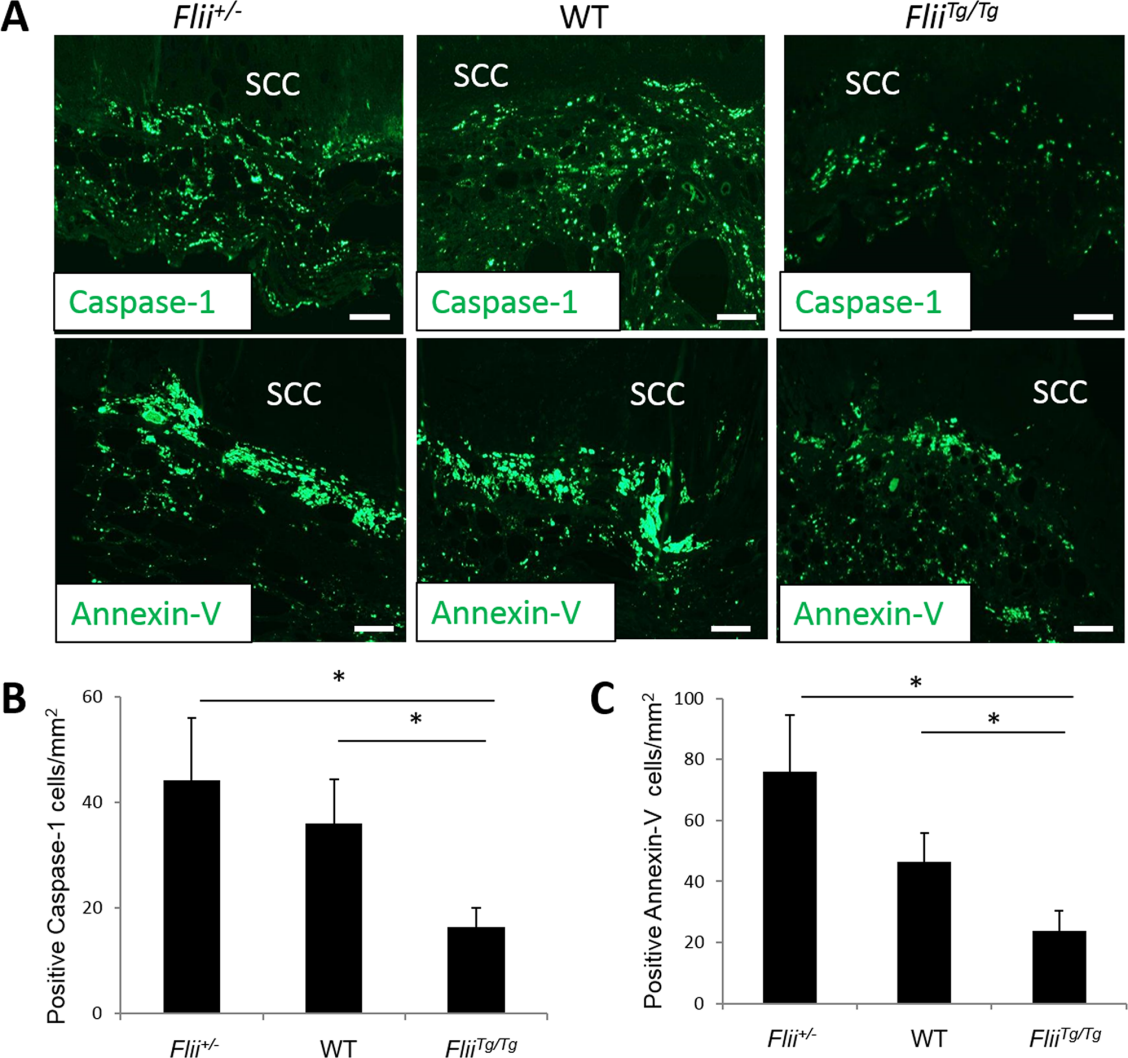
High levels of Flii lead to an evasion of apoptosis in cutaneous SCC tumors **A–C.** Cutaneous SCC tumors in *Flii^+/−^*, WT and *Flii^Tg/Tg^* mice were stained for caspase-1 and Annexin-V markers of apoptosis and number of positive cells analysed showing significantly decreased levels of apoptosis in tumors of *Flii* overexpressing mice. (*n* = 12/genotype). Magnification ×20. Scale Bar = 100 μm. Mean +/− SD *P < 0.05.

Cutaneous SCCs induced in *Flii^+/−^*, wild-type and *Flii^Tg/Tg^* mice were stained for markers of proliferation (PCNA (Figure 3A–3B)), and apoptosis (Caspase-1, Annexin-V (Figure 4A–4C)). No differences were observed in the numbers of PCNA positive proliferating cells between the tumors originating from the *Flii^+/−^*, wild-type or *Flii^Tg/Tg^* mice suggesting that Flii is not directly affecting cell proliferation in these tumors. (Figure 3A–3B). Caspase 1 and Annexin-V were significantly decreased in *Flii^Tg/Tg^* SCCs (Figure 4A–4C) compared to wild-type and *Flii^+/−^* mice tumors suggesting that high Flii levels may aid in tumor evasion of apoptosis in Flii overexpressing mice.

### Exogenous reduction of Flii decreases tumor size and progression

**Figure 5:**
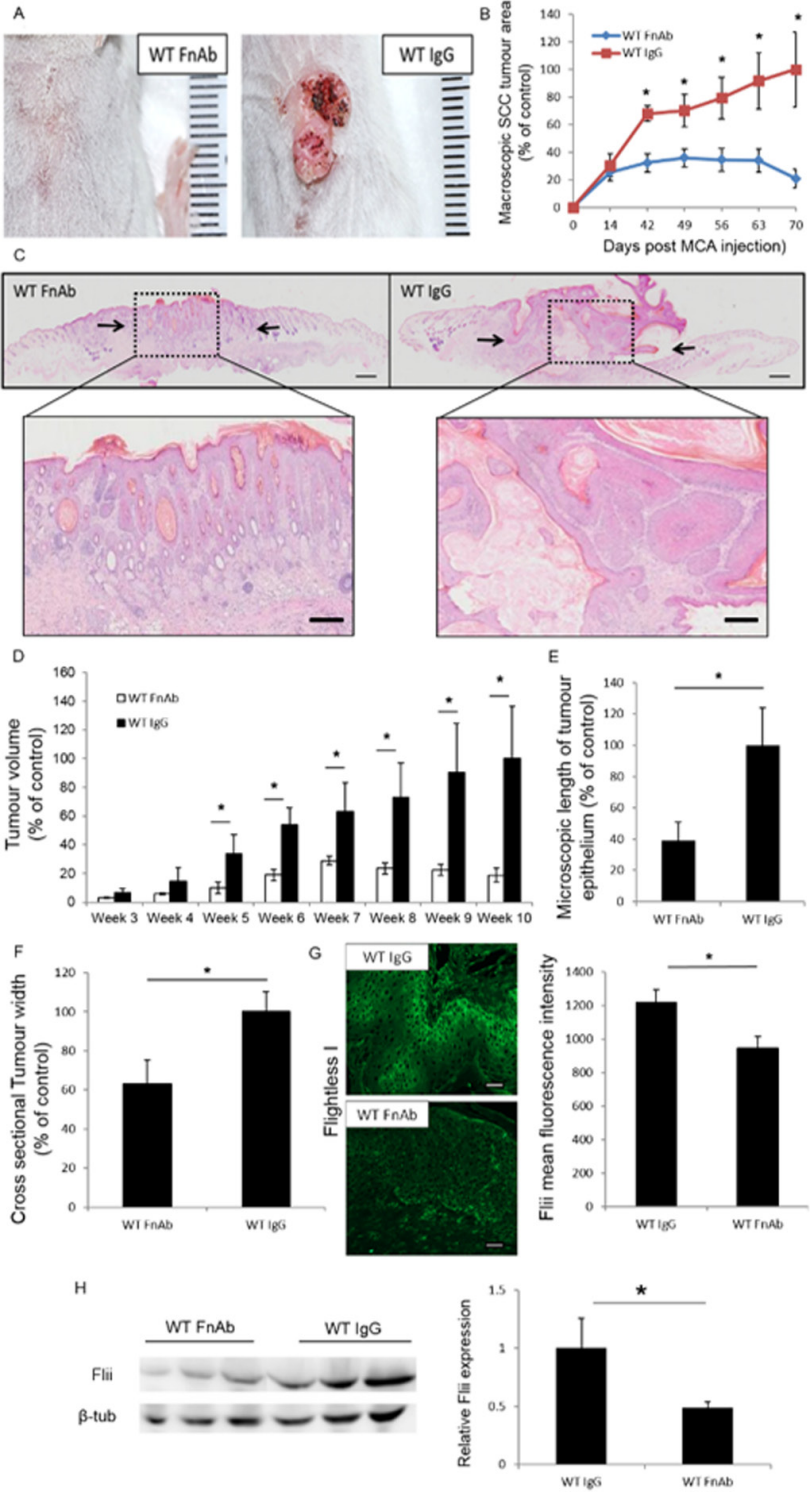
Reducing Flii expression during SCC initiation and development using FnAb results in decreased SCC progression WT mice were treated with FnAb at time of SCC induction and every second week throughout the trial. **A–B.** Examining the effect of reducing Flii levels using FnAb at time of SCC induction and during development resulted in decreased tumor progression and delayed development with significantly smaller tumor volume. (*n* = 12/treatment) Mean +/− SD *P < 0.05. **C.** Representative images of H&E stained tumor sections treated with FnAb and IgG control show smaller tumor size and less advanced tumor histology with less invasion, outgrowth and hyperkeratotic nodule formation. Magnification ×4 Insert ×20. Scale Bar = 500 μm and 100 μm. **D–F.** Macroscopic analysis of tumor volume and microscopic histological analysis of tumors revealed significantly decreased tumor volume, length of tumor epithelium and cross sectional tumor width in FnAb treated tumors. (*n* = 12/treatment) Mean +/− SD *P < 0.05. **G.** Representative images and graphical representation of significantly reduced Flii expression in FnAb treated tumors compared to IgG treated controls. (*n* = 12/treatment) Mean +/− SD *P < 0.05. **H.** Western Blot analysis of Flii levels in serum of FnAb treated and IgG control SCC induced wild-type mice showing significant reduction in Flii serum levels following FnAb treatment. (*n* = 3/treatment) Mean +/− SD *P < 0.05. Figure representative of three experimental repeats.

Intradermal injections of Flii neutralizing antibodies (FnAb) to the tumor site of WT mice at week 0, 2, 4, 6 and 8 decreased the tumor size and volume compared to control injections of irrelevant IgG antibody (Figure 5A). The volume of the tumors was measured macroscopically at weekly time points with control-treated tumors increasing up to 5 fold at week 10 in both tumor area and volume compared to FnAb-treated tumors (Figure 5B–5D). Histological analysis confirmed that FnAb treated tumors were significantly smaller with decreased length of tumor epithelium and cross sectional tumor width (Figure 5E–5F). They further showed less invasion, reduced cellular outgrowths and limited hyperkeratotic nodule formation (Figure 4C). Flii levels were significantly reduced in FnAb treated tumors (22.50% +/− 7.53% reduction) and serum (51.57% +/− 5.01% reduction) of FnAb treated mice compared to control treated tumors and serum of control mice respectively (Figure 5G–5H).

### Exogenous reduction of Flii prior to SCC induction decreases SCC development

**Figure 6:**
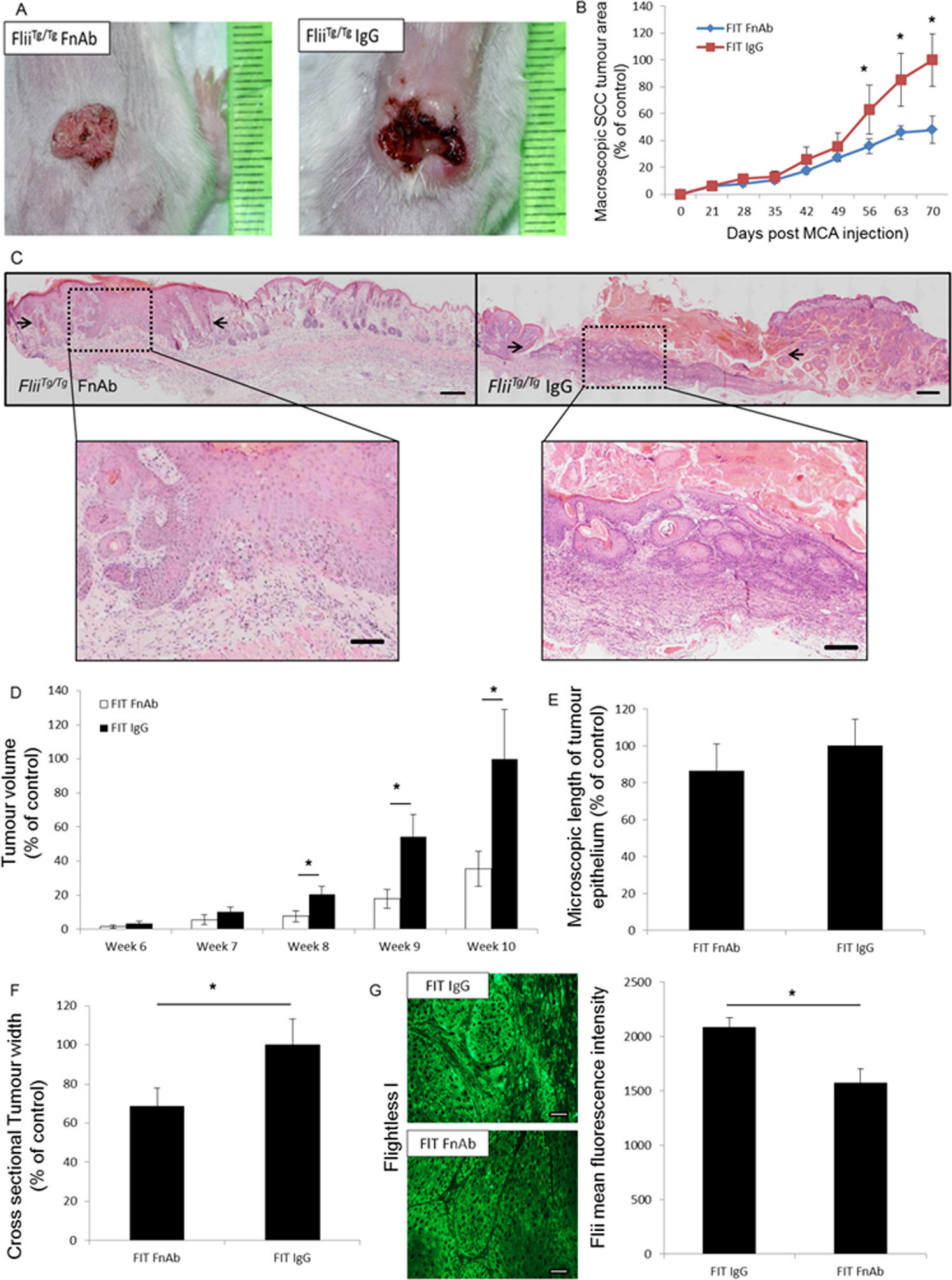
Reducing Flii expression prior to SCC initiation and development using FnAb results in decreased SCC progression As Flii is increased in human SCC samples, effect of preventative FnAb treatment prior to SCC induction was investigated in *Flii^Tg/Tg^* mice. Mice were treated with FnAb two weeks prior to SCC induction and every second week throughout the trial and SCC development. **A–B.** Examining the effect of reducing Flii levels in mice skin using preventative FnAb treatment prior to SCC induction and during development resulted in decreased tumor progression and size in FnAb treated mice. IgG control mice have significantly larger and more developed necrotic ulcerated tumors. (*n* = 12/treatment) Mean +/− SD *P < 0.05. **C.** Representative images of H&E stained tumors treated with FnAb or IgG control show more severe ulcerated tumor pathology in IgG control group (black arrows). Magnification ×4 Insert ×20. Scale Bar = 500 μm and 100 μm. **D–F.** Macroscopic analysis of tumor volume and microscopic histological analysis of tumors revealed no significant difference in length of tumor epithelium however significantly smaller cross tumor volume and cross sectional tumor width was observed in FnAb treated tumors. **G.** Representative images and graphical representation of significantly reduced Flii expression in FnAb treated tumors compared to IgG treated controls. (*n* = 12/treatment) Mean +/− SD *P < 0.05.

Flii is elevated in SCCs of human and murine origin (Figure 1) and increased expression of Flii escalates the size and progression of tumor formation (Figure 2). Flii levels are elevated in *Flii^Tg/Tg^*mice therefore to determine if reducing the level of Flii prior to SCC formation reduced the size and progression of tumors, FnAbs were intradermally injected into the dorsal skin of *Flii^Tg/Tg^*mice for two weeks prior to SCC initiation and then every two weeks until week 10 when the study was completed with the aim to maintain low levels of Flii within the skin. The resulting tumors of FnAb treated *Flii^Tg/Tg^* mice were significantly smaller than IgG controls and comparable to those of untreated wild-type mice (Figure 6A). Tumor area and volumes were up to 2.5 fold smaller than IgG treated mice (Figure 6B–6D). While microscopic analysis showed no significant difference in microscopic length of tumor epithelium (Figure 6E), FnAb treated tumors were confirmed to be significantly smaller with significantly smaller cross sectional tumor width (Figure 6F). *Flii^Tg/Tg^* FnAb treated tumors had significantly reduced Flii expression (24.53% +/− 8.35% reduction) compared to IgG treated controls (Figure 6G) and similar levels as observed in untreated wild-type SCC tumors (Figure 6G).

### Reducing Flii decreases SCC tumor sphere formation and keratinocyte invasion *in-vitro*

**Figure 7:**
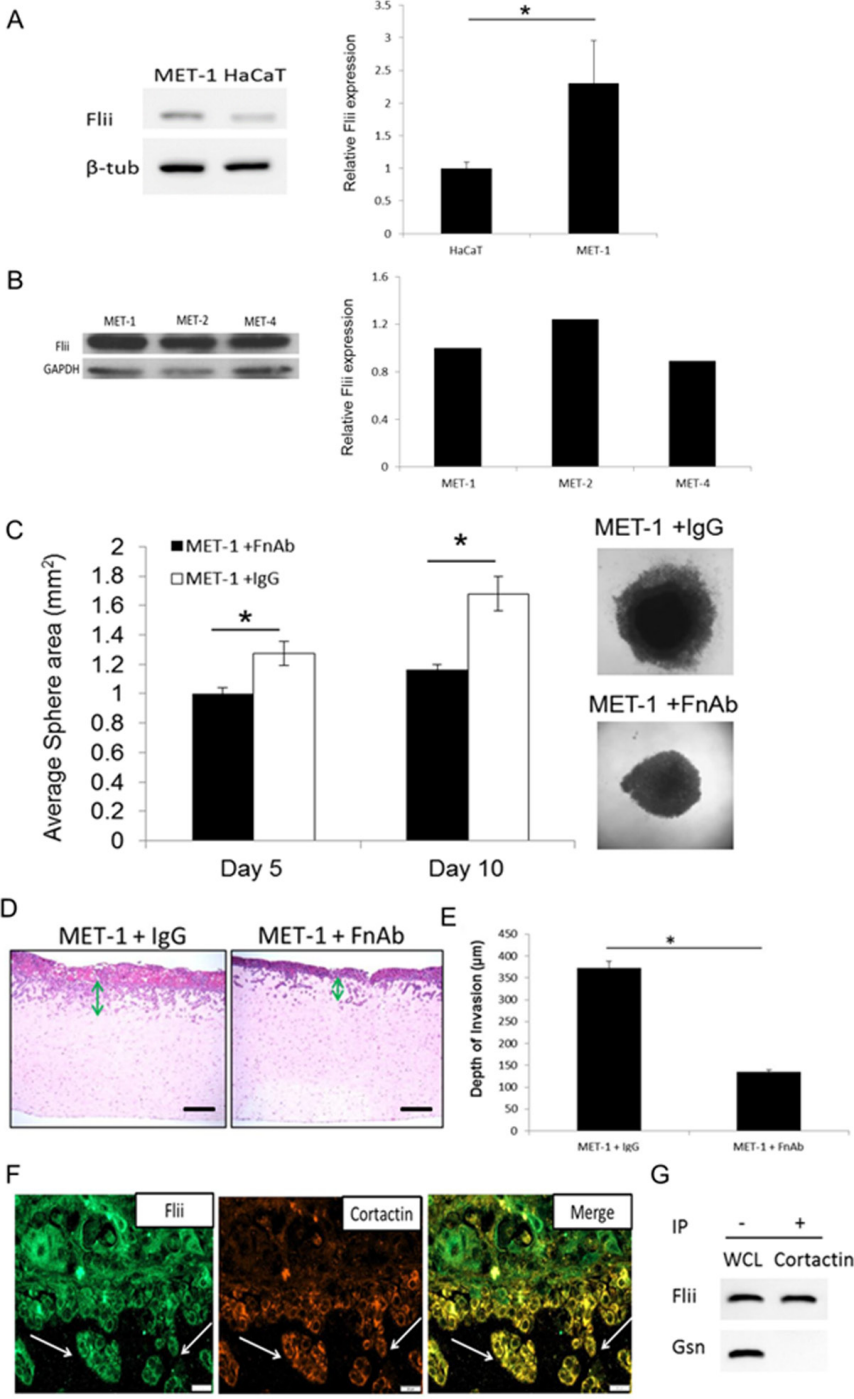
Reducing Flii expression using FnAb reduces sphere formation and invasion properties of SCC keratinocytes in-vitro Flii levels in MET-1 SCC cells were compared to both HaCaT immortalized keratinocyte cell line **A.** and MET-2 (cells from recurrent tumor of same patient) and MET-4 (cells from metastatic tumor of same patient) SCC cell line **B.** showing significant increase in Flii levels in MET-1 SCC cell line compared to HaCaT immortalized keratinocytes. **C.** The effect of reducing Flii using FnAb (50 μg/ml) or dose matched IgG antibody control on MET-1 SCC keratinocyte ability to form tumor spheres was analysed. MET-1 SCC keratinocytes treated with FnAb showed a significantly delayed ability and reduced sphere formation. Representative images of sphere size at day 10 are shown. *N* = 8. Mean +/− SD *p < 0.05. Scale bar = 500 μm refers to all images. Sporadic SCC cell line, MET-1, was used in a 3D organotypic model of cell invasion. Cells were treated with FnAb (50 μg/ml) at time of gel preparation and in media throughout the ten day period. **D–E.** Representative images of H&E stained gels illustrating cell invasion properties. Treatment of MET-1 SCC cells with FnAb significantly reduced the cell invasion properties (green arrow) into the gel matrix with 60% decreased depth of invasion. (*n* = 3/treatment) Magnification = 10×. Mean +/− SD *P < 0.05. **F.** Considering the function of Flii in actin remodelling, Flii association with invadopodia marker cortactin was examined using immunohistochemistry. Representative confocal images of dual immunofluorescence of MET-1 SCC cell lines shown Flii (green) was prominent throughout the epidermis highlighting all SCC cells, while cortactin (orange) was only prominent in invading cells highlighting the invadopodia. Flii co-localized with cortactin (yellow) suggesting a tumor promoting activity for Flii in SCC cancer cell invasion. Magnification = 400×. **G.** Anti-cortactin immunoprecipitate (IP) was prepared from confluent MET-1 SCC cell line and immunoblotted with Flii or Gelsolin (positive control) antibodies. Flii but not Gelsolin was found to associate with cortactin and specific bands were observed in MET-1 cell lysate (WCL) control. Data are representative of three independent experiments.

Flii levels were significantly elevated (130% +/− 66% increase) in MET-1 SCC cells compared to HaCaTs (Figure 7A). Flii levels in MET-1 SCC cells were further compared to MET-2 (cells from recurrent tumor of same patient) and MET-4 (cells from metastatic tumor of same patient) SCC cell lines (Figure 7B). No significant difference in Flii levels were observed between these different SCC cell lines. MET-1 SCC keratinocytes were assessed for the effect of FnAb on sphere formation. The effect of attenuating Flii on tumor microenvironment and sphere formation was determined with significantly smaller sphere areas observed at both day 5 and day 10 post seeding, when compared to IgG treated controls (Figure 7C). Additionally, using an organotypic model of cell invasion in three dimensions (3D), the effect of reduced Flii expression on MET-1 SCC keratinocytes was investigated *in-vitro*. Addition of FnAb resulted in a significant reduction in invasion of MET-1 SCC keratinocytes into the collagen/matrigel matrix compared to IgG treated controls (Figure 7D–7E) with a reduction of 63.81% +/− 1.92% observed (Figure 7D–7E). Immunofluorescence staining of the 3D organotypic gels showed that Flii (green) was present throughout the epidermis and co-localized with cortactin (orange) which is a marker of invadopodia. Flii and cortactin localized together in the tumor cells and islets invading into the dermis (yellow) (Figure 7F). Flii and cortactin were confirmed to associate using anti-cortactin immunoprecipitates derived from a confluent culture of MET-1 SCC keratinocytes and immunoblotted for Flii (Figure 7G). No association was observed between gelsolin and cortactin, despite robust expression of gelsolin in MET-1 cells suggesting a Flii specific association with the invadopodia marker (Figure 7G).

## DISCUSSION

Flii is an important inhibitor of cellular adhesion and a promoter of both actin remodeling and cell signaling with demonstrated functions in actin polymerization and nuclear receptor co-activation respectively [40, 41]. Flii colocalizes with actin and microtubule based structures [11], controls actin remodeling and polymerization [23, 42], stimulates integrin based cell migration [24] and controls cell adhesion to extracellular matrix substrates [25], all of which are instrumental in the ability of tumor cells to migrate and invade surrounding tissue. Here we have shown that Flii promotes SCC tumor development via its effects on tumor apoptosis and invasion and that delayed onset and decreased progression of SCC can be achieved by reducing Flii levels in tumors *in-vivo*.

Flii is increased in human and mouse SCC tissue samples, is elevated in the sera of patients with SCC and is increased in different SCC cell lines established from human primary, recurring and metastatic SCC as well as immortalized keratinocytes. *Flii* over expression resulted in larger, more aggressive tumors with an invasive tissue pathology. While no metastasis to secondary sites or perineural invasion was observed in this model of SCC, *Flii* overexpressing mice did show more differentiated tumor pathology compared to wild-type controls. Tumors in the *Flii* overexpressing mice had significantly decreased levels of early apoptotic markers annexin-V and pro-inflammatory caspase-1, potentially indicating reduced apoptosis occurring in these tumors which would increase SCC tumor progression. Indeed, previous studies have described the important suppressive function of Flii as an inhibitor of pro-inflammatory caspase activation and caspase-1 mediated apoptosis [21, 22]. This literature and our results together suggest that the inhibitory effects of Flii on caspase-1 activation and apoptosis may aid SCC tumor evasion in *Flii^Tg/Tg^* mice. In contrast, *Flii* heterozygous mice developed less invasive tumors similar to those observed in wild-type control mice and showed no differences in tumor cell proliferation or apoptosis compared to control animals. While Flii levels are reduced in heterozygous mice they still retain 50% of gene expression which may be sufficient for up-regulation of Flii to levels required to initiate tumor formation, however these mice did show significantly smaller tumor area and decreased length of tumor epithelium compared to both wild-type and *Flii* overexpressing mice. The mouse model of SCC used in this study is based on a single intradermal injection of the carcinogen MCA and has been used in many SCC studies [43–45]. However, our observations may be model specific particularly as this model has the potential to induce sarcomas if the injection is sub-cutaneous rather than intradermal. [45].

Reducing Flii expression in SCC tumors *in-vivo* using intradermal injections of FnAb resulted in significantly decreased Flii levels in both the tumor microenvironment and in the serum and led to significantly smaller tumors, both macroscopically and microscopically suggesting that reducing Flii may aid in delaying tumor formation and/or progression. In agreement with these studies, FnAb has previously been shown to neutralize Flii activity *in-vitro* and *in-vivo* [35, 46] and can deplete Flii present in conditioned media by over 90% [47]. No studies to date have investigated the function of extracellular Flii in tumorigenesis however our findings of increased Flii levels in invading tumor cells as well as decreased Flii serum levels and tumor growth following FnAb treatment suggest that Flii may promote tumor development and progression. Secretion of gelsolin family members into the circulation has previously been linked to functions in scavenging actin during tissue injury while Flii secretion has been associated with tissue injury and regulation of chronic inflammation [28, 48, 49]. The pathway of Flii secretion has been characterized [47] but to date no Flii cell surface receptors or pathways describing Flii impact have been described. Nonetheless, FnAb has been shown to be taken up into cells [31] so potentially it may neutralize both extracellular and intracellular functions of Flii during SCC tumorigenesis.

Remodeling and polymerization of actin filaments and their associated actin-binding proteins is important in the formation of invadopodia and subsequent tumor cell migration and invasion [50]. Increased Flii leads to weaker cell-stroma and cell-cell adhesions with altered GTPase and Src signaling pathways which may favour tumor cell invasion into surrounding tissue [25]. In addition, previous studies have demonstrated a function for Flii in inhibiting integrin facilitated cell migration, with diffuse arrangement of integrin receptors in Flii overexpressing keratinocytes contributing to increased tumor cell invasion [24]. The tumor promoting activity associated with Flii may therefore be due to its effects on cell adhesion and migration and its effects on tumor-microenvironment. A significant decrease in tumor cell aggregation/sphere formation and matrix invasion was observed following treatment with FnAb *in vitro*. Flii also specifically associated with cortactin, a marker of invadopodia. Considering its function in actin remodeling and polymerization [23, 41] and its identification as one of the proteins present in the invadopodia complex, these findings suggest that Flii may promote tumor progression, and facilitate invadopodia formation and tumor invasion.

Our findings therefore suggest that Flii, like other members of the gelsolin family of proteins, can influence SCC tumor growth and progression via its effects on tumor invasion and apoptosis. These studies identify Flii as a novel protein involved in tumor progression that may be a target for the development of new tumor therapies.

## MATERIALS AND METHODS

### Antibodies and reagents

Mouse monoclonal anti-Flii (sc-21716), anti-cortactin E-4 (sc-55888) and anti-vimentin (V9) (sc-6260), rabbit polyclonal PCNA (FL-261) (sc-7907), Caspase-1 (M-20) (sc-514), Annexin-V (FL-319) (sc-8300) antibodies and 4′,6-Diamidino-2-phenylindole dihydrochloride (DAPI) were obtained from Santa Cruz Biotechnology (Santa Cruz, CA, USA). Murine IgG irrelevant (I8765), β-tubulin (T0198) and cytokeratin 14 (HPA000452), albumin (AO433) antibodies and normal human serum (H3667) were obtained from Sigma-Aldrich (Castle Hill, NSW, Australia). Alexa-Fluor 488 goat anti-rabbit (A11008) and Alexa-Fluor 594 goat anti-mouse (A11020) antibodies were obtained from Invitrogen Australia (Waverley, NSW, Australia). Gelsolin antibody (610412) was obtained from BD Biosciences, NSW, Australia. Mouse monoclonal anti-cytokeratin antibody (M0821) was obtained from Dako Australia, NSW, Australia. Affinity-purified mouse monoclonal anti-Flii (FnAb) IgG1 antibody raised against the Leucine-Rich Repeat (LRR) domain of Flii protein was made in our laboratory. 3-Methylcholanthrene (MCA) (213942) used to induce SCC was obtained from Sigma-Aldrich (Castle Hill, NSW, Australia).

### Cell lines

The human cutaneous invasive SCC keratinocyte cell lines from primary tumor (MET-1), reoccurring tumor (MET-2) and malignant lesion (MET-4) was kindly obtained from Prof Proby and was tested, authenticated and characterized by *in vitro* methods as previously described [51]. MET-1 cell line originally obtained from the forehead skin of the invasive primary tumor in an adult immunosuppressed patient [51] was cultured in DMEM:Ham's F12 (3:1) supplemented with 10% FCS, 1% L-glutamine (200 mM) and Ready Mix Plus (0.4 μg/ml hydrocortisone, 5 μg/ml insulin, 10 ng/ml EGF, 5 μg/ml transferrin, 8.4 ng/ml cholera toxin and 13 ng/ml liothyronine). The MET-1 cell line was derived from primary cutaneous SCC and spontaneously immortalised in culture, as previously described [51]. Human foreskin fibroblasts (HFFs) and human immortalized keratinocytes (HaCaTs) (Cell Lines Service, DKFZ, Heidelberg, Germany) were cultured in DMEM supplemented with 10% FCS and 1% L-glutamine (200 mM). All cells were cultured at 37°C with 5% CO_2_.

### Human tissue samples

Investigation has been conducted in accordance with the ethical standards and according to the Declaration of Helsinki and according to national and international guidelines and has been approved by the authors' institutional review board. Human SCC tumors (*n* = 21) were obtained from SA Pathology under oversight of the Royal Adelaide Hospital Research Ethics Committee (approval number 120113). Human SCC serum (*n* = 3) samples were kindly donated by A/Prof Ian Darby, RMIT University, Victoria, Australia under oversight of the St Vincent's Hospital Research Ethics Committee (approval number HREC-A 129/09). The clinical investigations were conducted according to the Declaration of Helsinki principles and written informed consent was obtained. The control group for immunohistochemistry experiments consisted of human skin samples collected during cosmetic surgical procedures (ethics approval number 11-CHREC-F007) from four patients with no known dermatological conditions. Normal human serum (H3667) obtained from Sigma-Aldrich (Castle Hill, NSW, Australia) was used as a control for western blotting experiments using SCC patient serum.

### Animal studies

Mice were maintained according to Australian Standards for Animal Care under protocols approved by the Child, Youth and Women's Health Service Animal Ethics Committee or the SA Pathology/Central Adelaide Local Health Network Animal Ethics Committee. All strains were BALB/c or FVB/n congenic and were maintained as homozygous colonies or by continuous backcross with animals of the relevant pure strain. Wild-type controls were obtained from BALB/c inbred litters. The murine alleles of Flii used in this study were (1) *Flii tm1Hdc* (MGI:2 179 825), a heterozygous carrier of this allele is written as *Flii*^+^*/*^−^; and (2) Tg (FLII)1Hdc (MGI:3 796 828), a transgenic strain expressing exogenous human *FLII* due to the insertion of the human cosmid clone c110H8, carriers have two copies of the mouse *Flii* gene and two copies of the human *Flii* transgene (*Flii*+*/*+; *FliiTg/Tg*) denoted throughout this article as *Flii^Tg/Tg^* [39].

### *In vivo* mouse model of SCC

Cutaneous SCC was induced in 12 week old adult female Balb/c *Flii^+/−^*, wild-type and *Flii^Tg/Tg^* mice (*n* = 12 time-point/treatment) following a previously published model of cutaneous SCC [44]. Briefly, a single intradermal injection of 3-Methylcholanthrene (MCA) (500 μg MCA in 100 μl of corn oil) was used to induce primary cutaneous SCC. Necrotic and ulcerated SCC developed over a 12 week period. At week 12 the mice were euthanized and tumor samples collected. SCCs were analyzed throughout the trial and at the endpoint by a) weekly macroscopic digital images of lesions; b) tumor volume measurements using electronic calipers; c) histological analysis of tumor invasion and tumor size and d) immunofluorescence analysis of Flii, cytokeratin and vimentin expression. Additionally, the effect of intradermally administered FnAb on SCC progression in WT and *Flii^Tg/Tg^* mice was performed. Four intradermal injections (25 μl) of 100 μl FnAb (50 μg/ml) or IgG control were injected into the MCA injection site or surrounding the tumor base as it developed at 0, 2, 4, 6 and 8 weeks (treatment trial) or −2, −1, 0, 2, 4, 6 and 8 weeks (prevention trial) (*n* = 12/treatment). Due to the increased SCC tumor growth and development observed in *Flii^Tg/Tg^* mice in the first trial in all subsequent experiments mice were euthanized at week 10 of these experiments and tumor samples collected.

Multi-stage cutaneous carcinogenesis was also carried out as previously described [8]. Briefly, dimethylbenz[a]anthracene (DMBA, Sigma, 25 μg) in acetone (150 μl) was applied directly to shaved dorsal skin. After three days, 12-O-tetradecanoylphorbol-13-acetate (TPA, Sigma, 6.25 μg) was applied in acetone (150 μl) and the application of TPA repeated twice weekly for 20 weeks or until an invasive carcinoma developed as determined by the conversion of pre-malignant papillomas of convex shape to a more concave appearance. Mice exhibiting carcinomas were humanely killed and the tumors dissected out for histological and immunohistochemical analysis.

### 3D organotypic invasion assay

The effect of FnAb on cellular invasion was investigated using the sporadic SCC cell line, MET-1, in organotypic cultures of collagen:matrigel gels as previously described [52]. Briefly, collagen:matrigel gels were prepared by mixing 3.5 volumes of type I collagen (First Link, UK), 3.5 volumes of Matrigel (BD Biosciences, UK), 1 volume of 10× DMEM, 1 volume of 10% FCS and 1 volume of DMEM with 10% FCS containing 5 × 10^6^/ml HFFs. FnAb (50 μg/ml) or control IgG (50 μg/ml) were added to the gel mixture. One ml of the gel mixture was placed into each well of a 24-well plate and allowed to polymerize at 37°C for 1 hour. After polymerization, 1 ml of DMEM was added per well and gels were incubated for 18 hours to equilibrate. MET-1 SCC keratinocytes were then seeded into a plastic ring placed on the top of the gel at a density of 5 × 10^5^ cells per gel. After 24 hours, the rings were removed and the gels raised to air-liquid interface on stainless steel grids. Media containing FnAb or dose matched control IgG (50 μg/ml) was changed every second day and the gels were harvested at day 10, fixed in 4% paraformaldehyde (PFA) and embedded in paraffin wax. Histological sections (4 μm) were stained with haematoxylin and eosin (*n* = 3/treatment), imaged by light microscopy and the depth of cellular invasion quantified using Image Pro-Plus computer software (Media Cybernetics Inc., Maryland, USA).

### Tumor sphere formation assay

The effect of FnAb on MET-1 SCC tumor sphere formation was assessed as previously described [53]. Briefly, MET-1 SCC cells were seeded in 96-well plates coated with 1.5% agarose (Sigma Aldrich, #A3768) at 5 × 10^3^ cells per well in complete media containing either FnAb (50 μg/ml) or IgG control (50 μg/ml). Media containing FnAb or IgG was changed every second day and phase contrast images of tumor spheroids captured at day 5 and 10 post seeding. Tumor sphere area was quantified using Image Pro-Plus computer software (Media Cybernetics Inc., Maryland, USA).

### Immunohistochemical analysis

Histological sections (4 μm) from paraffin-embedded fixed human healthy normal skin, human SCC tumors, murine MCA-induced *Flii^+/−^*, *Flii^+/+^* and *Flii^Tg/Tg^* SCC tumors, MET-1 FnAb or IgG treated organotypic gels or murine DMBA/TPA induced SCC tumors were used for immunofluorescence analysis following antigen retrieval according to the manufacturer's protocols (DAKO Corporation, Glostrup, Denmark). Following blocking in 3% normal horse serum, primary antibodies against Flii, cytokeratin 14, PCNA, CD31, VEGF, CD4, CD8, FOXP3, caspase-1, annexin-V and cortactin were applied at 1:100 and slides were incubated at 4°C overnight in a humidified chamber. The samples were subsequently washed 3 × 2 min in PBS before application of species specific Alexa Flour-488 or Alexa Flour-594 secondary antibodies (Invitrogen, Australia) for 1 hr at room temperature. Sections were washed 3 × 2 min in PBS followed by incubation with DAPI (1:5000) for 3 min at room temperature. Finally, slides were washed in PBS and mounted in Fluorescent Mounting Medium (Dako, Australia). Fluorescence intensity per unit area was determined using AnalySIS software (Soft Imaging System GmbH) and optical fluorescence analyzed in epidermis of the skin. InSpeck Microscope Image Intensity Calibration Kits (Invitrogen, Carlsbad CA) were used to define fluorescence intensity for constructing calibration curves and evaluating sample brightness. Area coverage analyses in epidermal cells within normal and tumor samples were conducted by first masking on cytokeratin 14 positive cells (red channel) and measuring area coverage within this area in the green (Flii) channel as previously described [7, 8]. Negative controls included replacing primary antibodies with normal rabbit IgG, or normal mouse IgG. For verification of staining, non-specific binding was determined by omitting primary or secondary antibodies. All control sections had negligible immunofluorescence.

Histological sections (4 μm) from paraffin-embedded fixed murine SCC-induced *Flii^+/−^*, *Flii^+/+^* and *Flii^Tg/Tg^* tumors, were used for immunohistochemical analysis of cytokeratin to confirm epithelial tumor origin following protocols previously described [44, 54]. Briefly, sections were quenched in 0.3% H_2_O_2_/methanol for 20 min prior to blocking in 3% NHS in PBS for 30 min. Primary antibody was applied to sections at 1:200 concentration in 3%NHS/PBS overnight in a moist air tight container at 4°C. Following PBS washes, species specific secondary biotinylated IgG secondary antibody was applied to sections at 1:200 concentration for 1 hr at room temperature. Following further PBS washes sections were subjected to the Vectastain ABC kit following manufacturer's protocol and expression level detected using Vector DAB (3,3′-diaminobenzidine) substrate. Images of cytokeratin stained tumors were analyzed using light microscopy.

### Western blotting

Protein was extracted from human serum samples and SCC induced or control mice serum samples, MET-1 −2, −4 SCC confluent keratinocytes or human immortalized HaCaT keratinocytes cell line following standard protein extraction protocols. Briefly, samples or cells were lysed in lysis buffer (50 mM Tris pH 7.5, 1 mM EDTA, 50 mM NaCl, 0.5% Triton X-100) containing protease inhibitor cocktail tablet (1 per 10 ml; Complete, Mini (Roche Products Pty Ltd, Dee Why, NSW, Australia)) and samples were homogenized briefly. Samples were centrifuged (16 000 × g, 10 min, at 4°C), with supernatants being transferred to fresh tubes and centrifuged for a further 10 min and the supernatants stored at −20°C. Sample protein (10 μg) was run on 10% SDS-PAGE gels at 100V for 1 hr and then transferred to nitrocellulose by wet transfer (Bio-Rad Laboratories, Regents Park, NSW, Australia) using standard Towbins Buffer with 20% Methanol at 100V for 1 hr. Membranes were blocked in 5% milk-blocking buffer for 10 min and primary antibodies added in PBS containing 5% skimmed milk powder, 0.3% Tween. Appropriate species-specific secondary horseradish peroxidase-conjugated antibodies were added for a further 1 hr at room temperature. Stringent washes for 1 hr were then performed before detection of horseradish peroxidase by Super Signal West Femto Maximum Sensitivity Substrate (Pierce Biotechnology, Rockford, IL) and capture using GeneSnap analysis program (SynGene, MD). Membranes were stripped and re-probed for β-tubulin (Sigma-Aldrich) for normalization of protein levels.

### Co-immunoprecipitation

Tissue lysate was extracted from MET-1 SCC keratinocytes following standard extraction protocols as described previously [24]. Briefly, confluent cells were lysed using a cell scraper and placed in a lysis buffer (50 mM Tris pH 7.5, 1 mM EDTA,50 mM NaCl, 0.5% Triton X-100) containing protease inhibitor cocktail tablet (1 per 10 ml; Complete, Mini (Roche, Australia)). Samples were centrifuged and supernatant containing cell lysate collected and used for co-immunoprecipitation. The tissue lysates were precleared by centrifugation, and incubated with 1 μg/μl cortactin antibody and protein G-agarose b (Invitrogen) overnight at 4°C. The immunoprecipitates were collected by centrifugation, pellets washed with lysis buffer, and stored in 2× sample buffer prior to standard Western Blotting analysis as previously described [24]. Western Blots were probed with Flii antibody or with Gelsolin antibody as a negative control. Whole cell lysate and beads alone were also loaded as experimental controls.

### Statistical analysis

Statistical differences were determined using the Student's *t*-test or an ANOVA. For data not following a normal distribution, the Mann-Whitney U test with Dunnet's post hoc test was performed. A *p* value of less than 0.05 was considered significant.

## SUPPLEMENTARY FIGURE


