# Supplementary material for: Cytoskeletal protein flightless I inhibits apoptosis, enhances tumor cell invasion and promotes cutaneous squamous cell carcinoma progression
Source: Oncotarget. 2015 Oct 19;6(34):36426–40. doi: 10.18632/oncotarget.5536 (PMC4742187; doi:10.18632/oncotarget.5536)
Supplement: Supplementary file 1 [file oncotarget-06-36426-s001.pdf]

SUPPLEMENTARY FIGURE

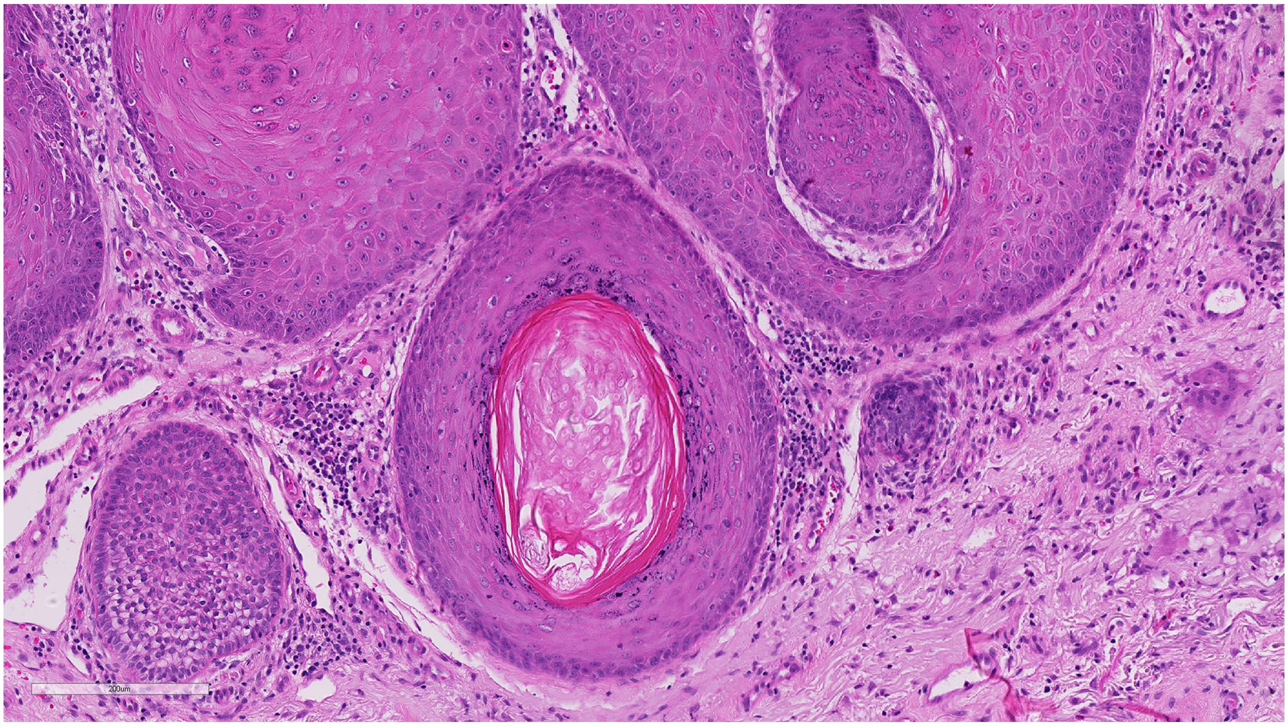

Supplementary Figure S1: Histological features of invasive cutaneous SCC in human biopsies. A. Representative image of the human SCC biopsy collected from a patient with invasive cutaneous SCC showing pathological features of invasive cutaneous SCC including: hyperplasia, immune infiltration, invasiveness and keratin pearls. Magnification = ×40. Scale Bar = 200 μm.
